# Similar, but different: drivers of the disproportionate HIV and sexually transmitted infection burden of key populations

**DOI:** 10.1002/jia2.25344

**Published:** 2019-08-30

**Authors:** Kenneth H Mayer, Lao‐Tzu Allan‐Blitz

**Affiliations:** ^1^ Beth Israel Deaconess Medical Center Harvard Medical School The Fenway Institute Boston MA; ^2^ Brigham and Women's Hospital Harvard Medical School Boston MA

**Keywords:** sexually transmitted infections, men who have sex with men, transgender people, sex workers, migrants, people who use drugs

Despite certain sexually transmitted infections (STI), for example, *Chlamydia trachomatis*, being sufficiently prevalent among the general population in some regions that they might be considered endemic, the contribution of “key populations” (KP) to recent increases in STI prevalence and incidence has been increasingly recognized 1. The definition of who belongs to a KP has varied among normative bodies, but common features include engagement in specific practices that augment risk (e.g. multiple partners, anal sex and/or sharing needles) and social marginalization, which can concentrate the partner pool because of limited opportunities to meet partners outside of risk milieu, while limiting access to needed treatment and prevention. The UNAIDS programme includes men who have sex with men (MSM), transgender people, sex workers, people who inject drugs (PWID) as KP 2 and incarcerated persons 3, 4, 5, 6, 7. Others have considered migrants to also be a KP 8, 9, 10, 11, given their disproportionate HIV/STI burden and lack of social protection. Addressing HIV diagnosis, treatment and prevention for KP is important for their individual health, as well as that of the wider community with whom they interact. Understanding the relationship of HIV spread between KP and others is often hindered by insufficient data.

Although members of KP sub‐groups may have different patterns of behaviour and social mixing that influence their HIV/STI risks, their vulnerabilities are augmented by common factors (Table 1). Often, KP experience structural barriers and societal discrimination that may increase their HIV/STI vulnerability by encumbering their access to healthcare 12, 13, 14, 15, 16, 17. Moreover, structural factors may not only directly affect susceptibility (e.g. lack of access to testing or treatment), but also shape behaviours and networks (e.g. being socially marginalized limiting partner choice). In settings where behaviours are criminalized 18, 19, 20, KP members may be at increased risk for HIV because of lack of access to condoms or sterile syringes, or may engage in avoidant behaviours due to the anticipation that insensitive providers might mistreat them 21, and fear of punitive action if they disclose unapproved sexual practices. KP avoiding healthcare are less likely to benefit from routine screening for HIV/STIs, early HIV/STI therapy (delaying the benefits of treatment as prevention, aka “TasP” for their partners), and/or pre‐exposure prophylaxis (PrEP). Internalized stigma and social ostracism have been linked to high rates of KP depression 22, 23, 24, anxiety and self‐medication with non‐prescription substances in order to alleviate distress 25, 26, 27, 28, which may further increase risky sexual practices. Their opportunities for gainful employment may be limited because of societal stigma, leading to sex work as their sole means of livelihood 29, 30. Financial incentives to engage in condomless sex, violence and lack of negotiating power exacerbate their vulnerability to HIV/STI.

**Table 1:** Multilevel drivers of enhanced susceptibility of key populations to HIV and other sexually transmitted infections^a^

Biology	Enhanced efficiency of anal intercourse Direct effects of acute STI (e.g. ulceration) Chronic mucosal inflammation due to multiple partners and sequelae of STI Microbial dysbiosis Role versatility (i.e. MSM and transgender women can be incentive or receptive partners)
Individual behaviour^b^	Depression, and other affective disorders (often due to internalized stigma) Substance use Avoidance of healthcare Condomless sex
Social networks	Number of partners/time Assortative mixing in high prevalence pools Sexualized venues (e.g. brothels, bathhouses, sex‐seeking social media)
Structural/institutional factors	Societal discrimination (e.g. growing up in non‐affirming environments) Health system discrimination (e.g. providers and health care institutions) Punitive and/or unsupportive laws (e.g. absence of anti‐discrimination protection) Criminalization Poverty Violence/victimization

^a^Men who have sex with men (MSM), transgender people, sex workers, people who inject drugs and migrants; Many of these factors are related to, and interact with, other factors depicted here; ^b^individual behaviours are often a direct or indirect response to structural factors.

Although there are common factors affecting HIV/STI vulnerability, some unique issues enhance transmission for some KP. Anal intercourse is extremely important in facilitating HIV/STI spread in MSM and transgender women, given that anal mucosa are particularly susceptible to HIV/STI acquisition and transmission 31, 32, and potentiating asymptomatic rectal STIs are common 33, 34. Although oral sex may be seen as an HIV risk reduction practice, it may potentiate the spread of other STIs, for example, *Neisseria gonorrhoeae* 35, 36, 37, 38. Natal males who engage in anal sex with other males have unique role versatility, since they can acquire infection through receptive intercourse, and then transmit as the insertive partner 39. Similar to enhanced transmission of HIV by sharing unsterile syringes, the risks posed by anal intercourse are addressable through access to condoms and antiretrovirals for prevention.

Social networks play a major role in increasing the efficiency of HIV/STI spread 40, 41. Sex workers and their partners may be at increased risk for HIV/STI 29, 30, 42. The presence of sexualized venues such as brothels, bathhouses and sex‐seeking social media create specific environments where HIV/STI can be efficiently spread 43, 44. These physical spaces and/or online connections 45, 46, 47 may lead to rapid partner turnover, increasing the likelihood of HIV/STI transmission. In socially marginalized populations with high HIV/STI prevalence, the limited choice of new partners leads to increased risk through assortative mixing. This phenomenon has been well‐characterized in Black American MSM, who have been shown to not be sexually riskier than demographically matched White MSM 48. Yet, because they are more likely to have other Black MSM partners, due to decreased social mobility and structural racism, their likelihood of encountering HIV/STI with any new partner is greater than White MSM 49.

Comparing and contrasting the dynamics of HIV/STI spread in different KP sub‐groups can help to inform policy, providing insights about general and specific needs. Attention to human rights should be integrated into any intervention focusing on KP, including the promotion of the rights of all individuals to be entitled to access life‐saving care, without fear of stigma, criminalization, or punitive practices by authorities, peers or others 50, 51, 52. KP members need to believe that their local healthcare systems are beneficent, and that access to, and affordability of, services are optimized, if they are to be effectively engaged and adherent to key medications. Providers need to be educated to provide culturally competent care 53, 54. An increasing array of resources is available to facilitate this, for example, www.lgbthealtheducation.org. Punitive laws that criminalize specific sexual practices, sex work, injection drug use and other socially marginalized behaviours, need to be removed so that individuals do not avoid seeking healthcare services that may improve their health, and that of their partners and the general community 55. To effectively address the increasing rise of STIs in the era of TasP and PrEP, sexual health education needs to discuss anal and oral sex among KP in nonstigmatizing ways.

Each KP group has specific issues that should be addressed in order to optimize their sexual health. Community empowerment interventions among sex workers have been associated with increased condom use and a reduction in HIV risk 56, 57, 58, while legislation to facilitate gender affirmation may be more beneficial in reducing risk among TP 59, 60. Other interventions may be appropriate for multiple groups. For example, MSM, TP, sex workers and PWID may all benefit from education about the risk of HIV and STI transmission from anal intercourse, contemporary options for safer sex, the benefits of early initiation of antiretroviral therapy for HIV‐infected individuals, and PrEP for those at risk. Early initiation of antiretroviral therapy for HIV‐infected individuals, and PrEP for those at risk, can decrease HIV spread, but will not mitigate the risk for STIs. Thus, education about the role of condoms in reducing STI transmission remains important, and if condoms are not accepted, then routine STI screening should be promoted. Harm reduction remains a cornerstone of any initiative to decrease HIV/STIs among PWID.

In summary, no single factor is driving increasing STI and HIV rates among KP. Multiple biological, behavioural and structural factors compound one another to potentiate individual and group risk. Most of these factors are socially and legally embedded (e.g. homophobia and transphobia), which may be expressed differently in diverse societies; but the lack of acceptance impeding individual development may lead to reactive depression and/or substance abuse, increasing sexual risk. For such individuals, a multi‐pronged approach is necessary if HIV/STI control is to be achieved: first of all: the removal of punitive laws that drive KP away from seeking needed services [62], then complemented by the education of providers and policymakers to develop culturally competent programmes to address clinical issues specific to KP, in addition to individual level interventions. One size will not fit all KP groups or individuals, yet commonalities exist. Understanding the similarities and differences driving risk is needed to effectively address the disproportionate burden of HIV and STI among KP.

## Competing interests

KHM and LA‐B have no competing interests to declare.
